# A New Definition for Intracranial Compliance to Evaluate Adult Hydrocephalus After Shunting

**DOI:** 10.3389/fbioe.2022.900644

**Published:** 2022-08-01

**Authors:** Seifollah Gholampour, Bakhtiar Yamini, Julie Droessler, David Frim

**Affiliations:** Department of Neurological Surgery, University of Chicago, Chicago, IL, United States

**Keywords:** brain material, hydrocephalus, shunt, intracranial compliance, intracranial pressure, fluid–structure interaction, viscous component

## Abstract

The clinical application of intracranial compliance (ICC), ∆V/∆P, as one of the most critical indexes for hydrocephalus evaluation was demonstrated previously. We suggest a new definition for the concept of ICC (long-term ICC) where there is a longer amount of elapsed time (up to 18 months after shunting) between the measurement of two values (V_1_ and V_2_ or P_1_ and P_2_). The head images of 15 adult patients with communicating hydrocephalus were provided with nine sets of imaging in nine stages: prior to shunting, and 1, 2, 3, 6, 9, 12, 15, and 18 months after shunting. In addition to measuring CSF volume (CSFV) in each stage, intracranial pressure (ICP) was also calculated using fluid–structure interaction simulation for the noninvasive calculation of ICC. Despite small increases in the brain volume (16.9%), there were considerable decreases in the ICP (70.4%) and CSFV (80.0%) of hydrocephalus patients after 18 months of shunting. The changes in CSFV, brain volume, and ICP values reached a stable condition 12, 15, and 6 months after shunting, respectively. The results showed that the brain tissue needs approximately two months to adapt itself to the fast and significant ICP reduction due to shunting. This may be related to the effect of the “viscous” component of brain tissue. The ICC trend between pre-shunting and the first month of shunting was descending for all patients with a “mean value” of 14.75 ± 0.6 ml/cm H_2_O. ICC changes in the other stages were oscillatory (nonuniform). Our noninvasive long-term ICC calculations showed a nonmonotonic trend in the CSFV–ICP graph, the lack of a *linear* relationship between ICC and ICP, and an oscillatory increase in ICC values during shunt treatment. The oscillatory changes in long-term ICC may reflect the clinical variations in hydrocephalus patients after shunting.

## Introduction

Hydrocephalus is a debilitating central nervous system disorder that is found in approximately 11/100,000 adults (Isaacs et al., 2018). The overall failure rate of the shunt, which is one of the most common treatment methods for hydrocephalus patients, is 32% in adults (Reddy et al., 2011). In addition to the challenging and controversial efficiency of shunts (Gholampour et al., 2019), there are also unpredictable clinical outcomes and complications in hydrocephalus patients after shunting. The reason for these various complexities is controversial; therefore, finding a clinical index to evaluate shunted hydrocephalus patients is of great importance.

The amplitude, baseline, or waveform of intracranial pressure (ICP) does not necessarily change in all types of hydrocephalus cases (Heldt et al., 2019; Meager et al., 2019). The quantitative thresholds of ICP (Raksin et al., 2003) and transmantle pressure gradient are not definable for all hydrocephalus patients. In addition, hydrocephalus is not necessarily due to impairments in the cerebrospinal fluid (CSF) absorption mechanism (Hakim et al., 2001; Gangemi et al., 2008). Even changes in CSF volume (CSFV) in some types of hydrocephalus are not adequately significant (Chang et al., 1992; Shaw and Million, 2012). Hence, these indexes cannot be fully comprehensive to evaluate all hydrocephalic complexities during the treatment process. Intracranial compliance (ICC) is one of the most important and practical indexes that is considered as a representative of cranial adaptation capacity and CSF–brain interaction. Many studies have shown that ICC (CSFV change–ICP change) can be a comprehensive index for the clinical evaluation of hydrocephalus patients (Marmarou et al., 1975; Yau et al., 2002; Raksin et al., 2003; Mase et al., 2005). There is a widely held belief that the trend of CSFV–ICP graph and ICC changes for hydrocephalus patients in a short time interval are monotonic and uniform (Marmarou et al., 1975; Sklar and Elashvili, 1977; Szewczykowski et al., 1977; Avezaat et al., 1979; Shapiro et al., 1980; Raksin et al., 2003). The goal of this study was to introduce a new definition for the concept of ICC to assess the trend of CSFV–ICP graph and ICC changes over an extended period of time (until 18 months after shunt treatment).

One of the most prevalent issues in previous investigations was the use of invasive methods for ICC measurement (especially for measuring the denominator of the ICC formula (ICP change) (Raksin et al., 2003; Heldt et al., 2019; Kazimierska et al., 2021)). Eide et al., Mahr et al., and Shapiro et al. measured ICP invasively using the ICP-monitoring method (Shapiro et al., 1993; Mahr et al., 2016; Eide, 2017). They also tried to find a relationship between mean wave amplitude and the volume–pressure response of the brain tissue. Invasive methods for ICC measurement pose the risk of bleeding and infection. Hence, many studies have tried to estimate the equivalent of ICC indirectly based on the relationship of ICP and/or ICC with other noninvasive measurable parameters such as the optic nerve sheath diameter (Sekhon et al., 2014; Sahu et al., 2021), transcranial Doppler outputs (Bellner et al., 2004; Rockswold, 2004), the cranium characteristics (Russegger and Ennemoser, 1990; Ueno et al., 1998; Mascarenhas et al., 2012), ICP pulse waveform (Brasil et al., 2021), and cerebral blood flow characteristics (Bateman, 2000; Egnor et al., 2001; Balédent et al., 2004). However, these methods have limitations and errors in ICC measurement (Raksin et al., 2003; Kazimierska et al., 2021). Alperin et al. and Mase et al. et al. calculated ICP using the continuity equation and the Navier–Stokes equation noninvasively (Alperin et al., 2000; Mase et al., 2005; Mase et al., 2008). We also used these equations under the fluid–structure interaction (FSI) simulation in our previous studies to identify the long-term changes in velocity, pressure, flow rate, the Reynolds number, the Womersley number, and vorticities of CSF flow in noncommunicating hydrocephalus patients after shunting (Gholampour et al., 2017a; Gholampour, 2018). We also used this method in this study for the noninvasive calculation of ICP and consequently ICC under the new definition in adult patients with communicating hydrocephalus until 18 months after shunting.

## Material and Methods

### MRI Patient Outputs

**FIGURE 1:**
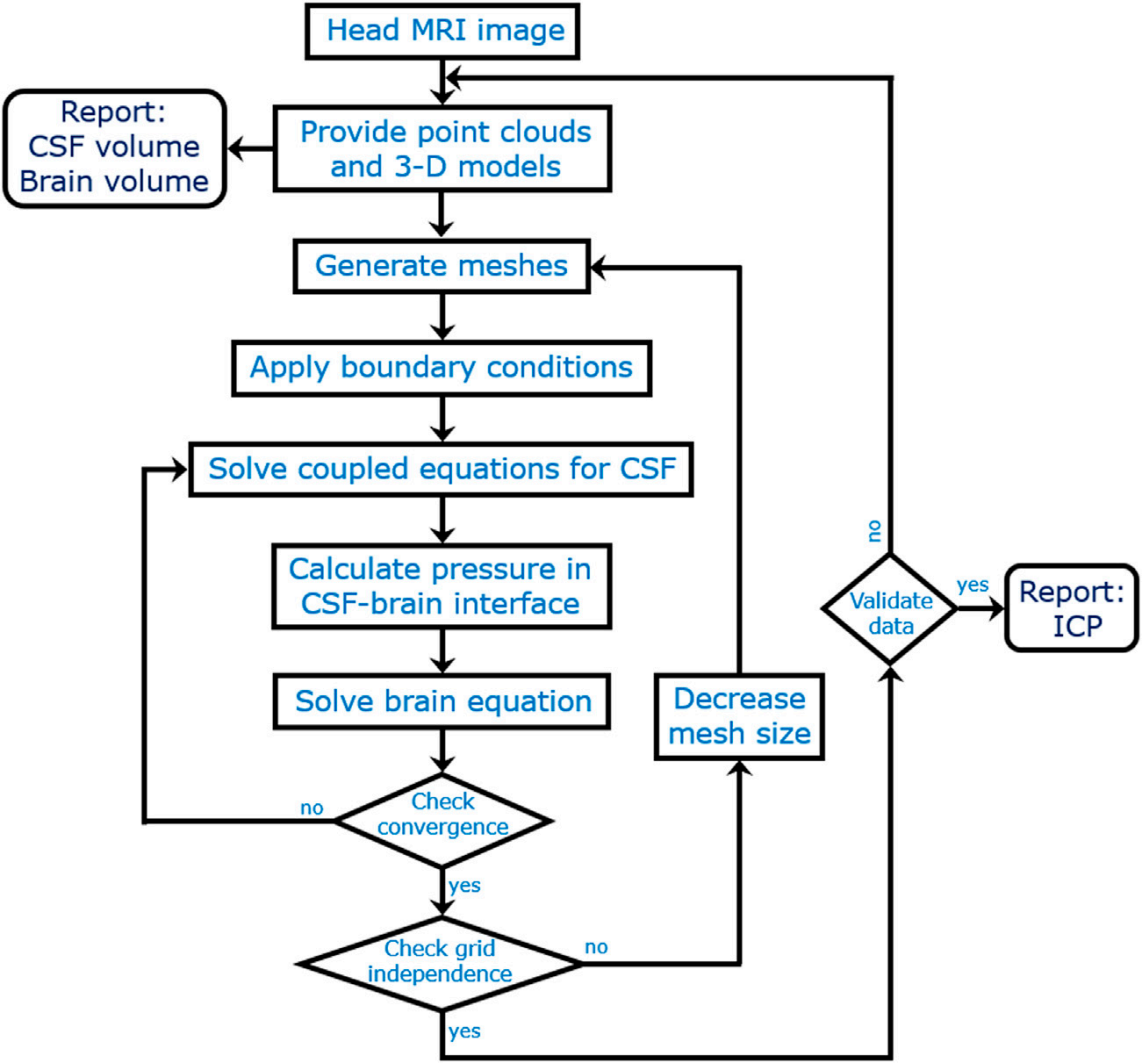
The methodological workflow.

**FIGURE 2:**
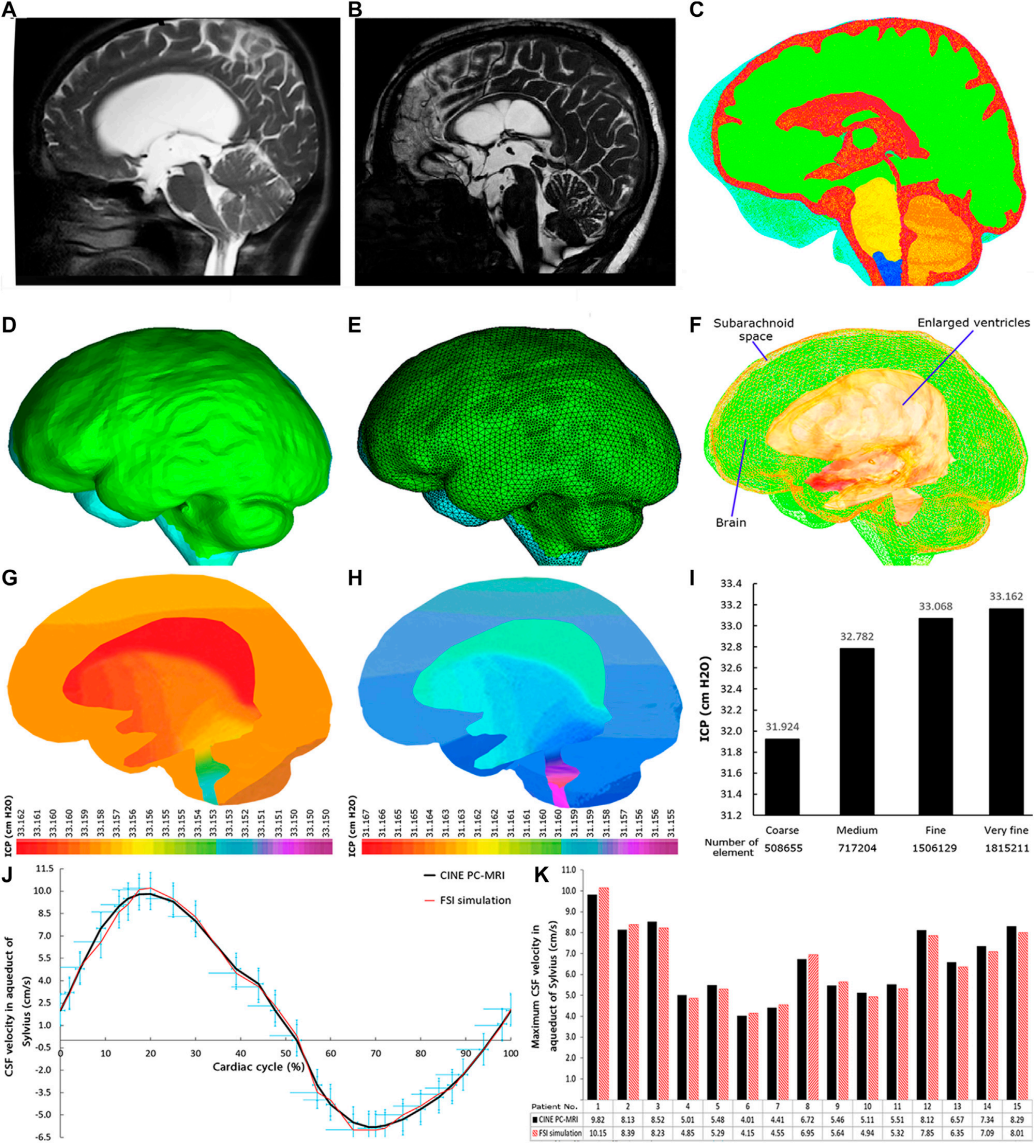
MRI, 3D geometrical model, meshed model, FSI simulation, grid independence study, and data validation. **(A)** and **(B)** show MRI of patient No. 2 pre-shunting and 12 months after shunting, respectively. **(C)** shows the head substructures in patient No. 2 18 months after shunting. The red and green areas show the fluid (ventricular system and subarachnoid space) and solid (brain tissue) domains, respectively. The borders and interfaces between red and green areas are FSI boundary conditions. **(D)** and **(E)** show the 3D geometrical model and meshed model of patient No. 2 prior to shunting, respectively. **(F)** shows the methodological workflow. **(G)** and **(H)** show two snapshots of ICP distribution at early systole and diastole phases, respectively, for patient No. 2 pre-shunting. **(I)** The grid independence study for the maximum ICP data. The difference between fine and very fine meshes in inpatient No. 2 prior to shunting was 0.28%. **(J)** The comparison of CSF velocity graphs in the aqueduct of Sylvius between CINE PC-MRI and FSI simulation data in a cardiac cycle for patient No. 1. **(K)** The comparison of the maximum CSF velocity in the aqueduct of Sylvius between CINE PC-MRI and FSI simulation data for all 15 patients. CSF: cerebrospinal fluid; FSI: fluid–structure interaction; CINE PC-MRI: cine phase-contrast magnetic resonance imaging; and ICP: intracranial pressure.

Among 39 adult patients with communicating hydrocephalus, 15 patients did not need shunt revisions and any changes in valve adjustment over the course of 18 months. This study is centered on ICC evaluations in hydrocephalus patients with shunt treatment to reach a healthy condition; hence, the patients with successful shunting were used in this study. The age and body mass index of the patients were 49–75 years and approximately 23.6–29.6 kg/m^2^. It should be noted that there was no history of other neurological problems in the patients used for this study. A Medtronic ventriculoperitoneal shunt was used to treat all patients. The methodological workflow of the present study is shown in Figure 1. Head cine phase-contrast magnetic resonance imaging (CINE PC-MRI) for 15 patients (seven females and eight males) was provided over the course of nine stages: prior to shunting, and 1, 2, 3, 6, 9, 12, 15, and 18 months after shunting (Figures 2A,B). The ethics committee of Tajrish Hospital, and the 1964 Helsinki Declaration and its later amendments approved the study design, procedures, and protocols. All patients provided written informed consent before undergoing any study-specific procedures.

All patients in each stage underwent MRI: a cardiac-gated PC flow quantification sequence and an axial T2-weighted image. The acquisition variables for PC-MRI include FOV = 160 × 160 mm, VENC = 15 cm/s, FA = 10°, TE = 7 msec, TR = 21 msec, ST = 6 mm, and acquisition time = 4.5 min. The acquisition variables for axial T2-weighted image include FOV = 220 × 220 mm, FA = 90°, TE = 117 msec, TR = 4,000 msec, ST = 6 mm, and acquisition time = 2.3 min. More information about the protocol is available in the study by Long et al. (2019). It should be noted that the MRI machine was a 3 T (MAGNETOM Trio; Siemens, Erlangen, Germany).

The method (modeling, formulation, simulation, constitutive model, and boundary conditions) that is used in this study is similar to our recent study (Gholampour and Fatouraee, 2021) that was utilized for healthy subjects and hydrocephalus patients prior to shunting. In this study, we repeated this procedure prior to shunting and eight stages after shunting. Nine CINE PC-MRI data (9 sets of imaging) for each patient (15 patients) amassed a total of 135 MRI data points that were provided for three goals: 1) CSF velocity graphs of patients in the aqueduct of Sylvius that are used for data validation; 2) blood flow rate graphs at the basilar artery are used for the model boundary conditions during FSI simulations; and 3) measurement of head substructure volumes (Figure 2C). To measure these volumes, DICOM files of head MRI were transferred to Mimics software (version 15; Tissueise, Leuven, Belgium) to create point clouds of each head substructure. The Mimics software, similar to previous studies, was used for manual segmentation of the brain tissue and CSF-filled spaces (ventricles and SAS) to measure volumes (Linninger et al., 2007; Linninger et al., 2009a; Sweetman et al., 2011; Sweetman and Linninger, 2011; Hsu et al., 2012). The point clouds then moved to the SolidWorks software (version 2018; Dassault Systemes SolidWorks Corp., Waltham, MA, United States) to create 3D geometrical models (Figure 2D) and measure the volumes. It should be noted that these 3D geometrical models were also transferred to the ADINA software (version 8.3; Adina R&D Inc., Watertown MA, United States) for both mesh generation (Figures 2E,F) and FSI simulation (Figures 2G,H). All procedures were repeated for all 9 imaging stages of the 15 patients. The methodological workflow is shown in Figure 2F.

### Computer Simulation Method

Despite recent advances in medical imaging technology, we are not able to measure ICP through imaging (Linninger et al., 2016; Gholampour et al., 2019). Even invasive methods such as ICP monitoring could not measure CSF pressure in all locations of the CSF circulation system. Therefore, in this study, computer simulation is used to calculate ICP noninvasively. Computational fluid dynamics (CFD) and FSI are two common computational methods to calculate ICP (Linninger et al., 2016; Gholampour et al., 2019). With regard to the deformable boundaries between CSF and the brain (specifically between CSF and the inner layer of the brain), CFD is not an accurate simulation method to calculate ICP (Linninger et al., 2009a; Sweetman et al., 2011; Gholampour and Fatouraee, 2021). Hence, similar to previous studies (Linninger et al., 2009a; Sweetman et al., 2011; Gholampour, 2018; Gholampour and Fatouraee, 2021), a two-way FSI with strong coupling was used based on arbitrary Lagrangian–Eulerian formulations (Adina, 2005) to calculate ICP values for each patient in each of the 9 stages. We had tested the method for noncommunicating hydrocephalus patients and healthy subjects by validating those results with experimental ICP values and *in vivo* CSF velocity values (Gholampour and Fatouraee, 2021). However, with regard to the approach of this study, we did not report the results of the healthy subjects in this study and used this method to calculate ICP of each patient in each of the nine stages. More details about the formulations and methods are explained in the study by Gholampour et al. (Gholampour and Fatouraee, 2021).

CSF as an incompressible Newtonian fluid and brain tissue were defined as fluid and solid domains, respectively. The law of continuity (conservation of mass) is defined for the CSF produced in the ventricular system and subarachnoid spaces (SAS) as shown in Eqs. 1 and 2, respectively. Equation 3 (conservation of momentum or the Navier–Stokes equation) was solved as a couple with the continuity equations (Eqs. 1 and 2) for the fluid domain. The coupled equations Eq. 1–4 governed the fluid and solid domains, respectively (Sweetman et al., 2011; Gholampour et al., 2017a; Gholampour et al., 2017b; Gholampour and Fatouraee, 2021).

$$\nabla .u_{F}=S$$ (1)

$$\nabla .u_{F}=0$$ (2)

$$\rho_{F}\frac{\partial u_{F}}{\partial t}+\rho_{F}\left(\left(u_{F}-W\right).\nabla \right)u_{F}=-\nabla \text{p}+\mu \nabla^{2}u_{F}+f_{F}^{B}$$ (3)

$$\nabla .\sigma_{S}+f_{F}^{B}=\rho_{S}{\ddot{u}}_{S}$$ (4)

Here, S, W, and u_F_ are the inlet CSF production, CSF velocity, and moving mesh velocity vectors, respectively. (u_F_-W) is the relative CSF velocity vector regarding the velocity of the moving coordinate. ρ_F_ and ρ_S_ are the densities of CSF and brain, respectively. $f_{F}^{B}$ is the body force per unit volume, and P is CSF pressure. µ, $\sigma_{S}$ , and ${\ddot{u}}_{S}$ are the CSF dynamic viscosity, brain stress tensor, and the local acceleration of the solid part, respectively. The dynamic viscosity and density of CSF were considered to be 0.001 kg m^−1^ s^−1^ and 998.2 kg m^−3^, respectively (Gholampour et al., 2017a; Gholampour et al., 2017b; Gholampour, 2018; Gholampour and Fatouraee, 2021).

### Constitutive Model of Brain

Hrapko et al. showed the time-dependent property of the viscous component of the brain tissue (Hrapko et al., 2006). Various models defined in previous studies include proelastic, viscoelastic, and hyperviscoelastic for modeling the brain tissue of hydrocephalus patients (Dutta-Roy et al., 2008; Gholampour et al., 2017a; Gholampour et al., 2017b). However, Cheng et al., Mehrabian et al., and Gholampour et al. used the theory of poroviscoelasticity to define a constitutive model for hydrocephalic brains, which has accurate conformity with their experimental results (Cheng and Bilston, 2010; Mehrabian and Abousleiman, 2011; Gholampour, 2018; Gholampour and Fatouraee, 2021). Derivation of the general equation was based on the equilibrium conditions of the stress, Darcy’s law of CSF flow in the porous medium, and the equation of the solid model (brain tissue) (Cheng and Bilston, 2010; Gholampour, 2018; Gholampour and Fatouraee, 2021). The viscoelastic component of the brain tissue was defined by the Prony series. Equation 5 expressed a time-dependent shear modulus of relaxation that was defined for this poroviscoelastic model (Cheng and Bilston, 2010; Gholampour, 2018; Gholampour and Fatouraee, 2021).

$$\text{Gr}\left(t\right)={\text{G}}_{0}\left(1-\overset{N}{\underset{k=1}{\sum }}g_{k}^{p}\left(1-e^{-\left(\frac{t}{\tau_{k}}\right)}\right)\right)$$ (5)

where G_o_ is the instantaneous shear modulus. Input parameters of the Prony series to dominate the response of the relaxation include τ _1_ = 3.1 (s), τ _2_ = 27 (s), τ _3_ = 410 (s), and $g_{k}^{p}$ = 0.285 (Cheng and Bilston, 2007; Cheng and Bilston, 2010; Gholampour et al., 2017b; Gholampour, 2018). The modulus of elasticity, permeability, Poisson’s ratio, and void ratio, which were used in the formulations, were 584.4 Pa, 4.08 × 10^−12^ M^4^/N.s, 0.35, and 0.2, respectively (Cheng and Bilston, 2010; Sweetman and Linninger, 2011; Gholampour et al., 2017b; Gholampour, 2018). It should be noted that these constant values were validated in previous studies (Gholampour, 2018; Gholampour et al., 2017b; Cheng and Bilston, 2010, Cheng and Bilston, 2007).

### Boundary Conditions

FSI simulation is very sensitive to boundary conditions (Gholampour and Fatouraee, 2021). After comparing all boundary conditions to simulate the CSF circulation system, our recent study showed that the most accurate inlet and outlet boundary conditions for simulation of hydrocephalus patients are the pulsatile CSF flow rate (Gholampour and Fatouraee, 2021). Despite the production of the CSF in the ventricular system and even SAS, with regard to the findings of previous studies (Linninger et al., 2007; Linninger et al., 2009a; Gholampour, 2018; Gholampour and Fatouraee, 2021), frontal horns of the lateral ventricles were considered as a location of the inlet flow during the modeling process. Although CSF will be absorbed in extracranial lymphatic pathways, in arachnoid granulations, and in additional intraparenchymal routes, the outlet places of the CSF are considered as the spinal cord and sagittal sinus (Gholampour, 2018; Gholampour and Fatouraee, 2021). Previous studies have shown that the most accurate inlet and outlets boundary conditions are the CSF flow rate graphs obtained by superposing a graph with a constant value (first graph) and a pulsatile graph (second graph) in the MATLAB software (version R2018; MathWorks, Natick, MA, United States) (Gholampour et al., 2017a; Gholampour, 2018; Gholampour and Fatouraee, 2021). The constant values of the first graphs for the inlet CSF flow, outlet CSF in the spinal cord, and outlet CSF in the sagittal sinus were 0.35, 0.17, and 0.18 ml/min, respectively (Gholampour, 2018; Gholampour and Fatouraee, 2021). The pulsatile graphs (second graphs) in one inlet and two outlet flow rate graphs were obtained from the normalized diagram of the “blood flow rate–time” curve in the basilar arteries, which were measured by CINE PC-MRI *in vivo* (the second goal of the MRI). The normalized diagram of “flow rate–time” represents a diagram that is independent of values (its vertical axis is from −1 to 1) and only reflects pulsatility. It should be noted that these inlet/outlet boundary conditions for each patient in each stage were calculated separately using the MATLAB software and applied as inlet/outlet diagrams in the ADINA software during the FSI simulation process.

It is worth mentioning that the 3D geometrical model of the head included the CSF model (ventricular system and SAS) and the solid model (brain) (Figure 2C). The recent study showed that the skull and dura mater (outer layer of the SAS) do not have a considerable effect on ICP calculations for hydrocephalus patients (Gholampour and Fatouraee, 2021). Hence, the outer layer of the SAS is constrained by a no-slip boundary condition. Interfaces between the inner and outer layers of the brain with CSF were defined as FSI boundaries (Figure 2C). Equations 6–8 indicated displacement compatibility between CSF and brain tissue (in the FSI interface), traction equilibrium between stresses acting in normal direction on both CSF and the brain domains, and the velocities in brain interfaces that are equal to the CSF velocity at the FSI interface (Gholampour et al., 2017a; Gholampour, 2018). These equations governed the FSI boundaries.

$$d_{S}=d_{F}\left(x,\;y,\;z\right)\epsilon \Gamma_{wall}^{F}\cap \Gamma_{wall}^{S}$$ (6)

$$\sigma_{S}.n=\sigma_{F}.n\;\left(x,\;y,\;z\right)\epsilon \Gamma_{wall}^{F}\cap \Gamma_{wall}^{S}$$ (7)

$$u_{S}=u_{F}\;\left(x,\;y,\;z\right)\epsilon \Gamma_{wall}^{F}\cap \Gamma_{wall}^{S}$$ (8)

Here, d_F_ and d_S_ are displacements of CSF and brain along with the FSI boundaries. $\sigma_{F}$ .n and $\sigma_{S}$ .n are the CSF and brain stress tensors, respectively, that are in the normal direction of the FSI interfaces, and u_s_ is the local acceleration of the solid part.

Our patients did not experience changes in the valve adjustment. Hence, we used the setting of the valve pressure that is used by neurosurgeons to simulate the outlet boundary conditions in the shunt tube. We defined a pressure diagram with maximum and minimum pressures equal 17.0 cm H_2_O and 14.5 cm H_2_O, respectively. It should be noted that the inner diameter of the tube equaled 1.3 mm and the location of the tip of the catheter in all patients was in the right lateral ventricle. The Medtronic shunt catalog is useful for more information about the values of outlet pressure boundaries and tube geometry: Medtronic, Strata Various Adjustment System, Minnesota, United States.

### Grid Independence Study

A tetrahedral (four-node) element was used for meshing the models. The differences between fine and very fine meshes in all 15 patients in all steps were less than 0.31% (Figure 2I). This result confirmed the mesh convergence process. The number of very fine meshes for patient No. 1 prior to shunting and 9 steps after shunting was 1,816,024, 1,781,450, 1,753,857, 1,720,118, 1,682,0137, 1,642,327, 1,593,109, 1,572,870, and 1,524,407, respectively. The implicit Euler scheme with a time step of 0.01 was used for refining the mesh grids, and smaller step sizes did not show differences in ICP results. It should be noted that the geometrical modeling and the FSI simulation process for all 9 steps of 15 patients took 2.5 years and several workstations with a 128-core server processor.

### Statistics and Reproducibility

Mean, standard deviation, standard error, coefficient of variation, confidence interval, and ANOVA tests were calculated by the IBM SPSS software (version 20.0; IBM Corp, Armonk, NY, United States). CSFV, brain volume, and ICP datasets governed a normal distribution based on the results of the Shapiro–Wilk test. All variances were the same based on the results of the homogeneity of the variance test. For pairwise comparison after the ANOVA test, Tukey’s post hoc test was used (Shim et al., 2019). We used Student’s t-test after ANOVA to compare CSF velocities measured by CINE PC-MRI and calculated by FSI simulation. The test statistics for ANOVA and Student’s t-test were F and T, respectively. Pearson’s correlation coefficient was used to describe the relationship between ICC and ICP. The results of the statistical analyses are listed in Supplementary Tables S1–S4. The statistical significance threshold is considered to be *p* <0.05. The values are described as mean value ± standard error.

## Results

### Data Validation

The validation of our assumptions of the FSI method (formulations, constitutive model for the brain tissue, and boundary conditions) was demonstrated in our previous study (Gholampour and Fatouraee, 2021). The most important advantage of this method is the conformity of the calculated ICP values with experimental ICP values for healthy and hydrocephalus patients, which were discussed in our previous study (Gholampour and Fatouraee, 2021). However, there is another common method in previous studies to validate simulation results. These studies compared the validity of one of the calculated results with that of *in vivo* or experimental data, because after finishing the simulation process and determining the correctness of grid indecency, all results will be calculated concurrently and under a common condition and method. In these studies, many parameters were calculated such as CSF velocity, stress, pressure, porosity changes, and CSF flow rate; however, they compared the validity of one of these calculated parameters with that of experimental or *in vivo* data. The simulation procedure is a unique process and method; hence, all parameters will be reported by the software under a unique and single “run” in each step. Therefore, one of these parameters will be able to reflect the accuracy of the method and assumptions, as well as all results. This approach has been used in previous computer simulation studies by comparing one of these parameters (CSF velocity) with CSF velocity measured by CINE-PC-MRI (Linninger et al., 2005; Linninger et al., 2009a; Linninger et al., 2009b; Sweetman et al., 2011; Sweetman and Linninger, 2011; Gholampour et al., 2017b). Therefore, CSF velocity in the aqueduct of Sylvius was measured for all 15 patients in 9 sets of imaging (prior to shunting, and 1, 2, 3, 6, 9, 12, 15, and 18 months after shunting) by CINE PC-MRI to compare with similar CSF velocities, which were calculated using FSI simulation. The phase lag between these CSF velocity graphs was less than 0.91% (Figure 2J). The differences between the maximum CSF velocities were less than 3.49% (Figure 2K). Necessarily, it does not mean our results have this error as sometimes the errors of the CINE PC-MRI method are also not negligible (Rutkowski et al., 2021).

In addition, we compared the correctness of our ICP results with that of ICP values of previous studies. The ICP values in previous communicating hydrocephalus patients prior to shunting were 26.80 cm H_2_O (Sweetman et al., 2011), 27.50 cm H_2_O (Linninger et al., 2009a), and 29.50 cm H_2_O (Linninger et al., 2007). The corresponding value in this study was 29.59 ± 0.8 cm H_2_O (Supplementary Table S3). The small differences between ICP values in these studies may be related to the differences in the intensity level of hydrocephalus in these patients.

In total, it is not very common to compare a patient’s CSFV with patients of a previous study, as each patient has unique conditions. However, the results of previous studies showed that CSFV (volume of ventricles and SAS) in communicating hydrocephalus patients prior to shunting were 371.1 ml (Linninger et al., 2007) and 410.2 ml (Sweetman et al., 2011). The corresponding value in this study was 416 ± 4.8 ml (Supplementary Table S1). The brain volume prior to shunting in the previous study (Sweetman et al., 2011) was 1,120.3 ml, and the corresponding value in this study was 1,050.5 ± 20.8 ml (Supplementary Table S2).

### Cerebrospinal Fluid Volume Change

**FIGURE 3:**
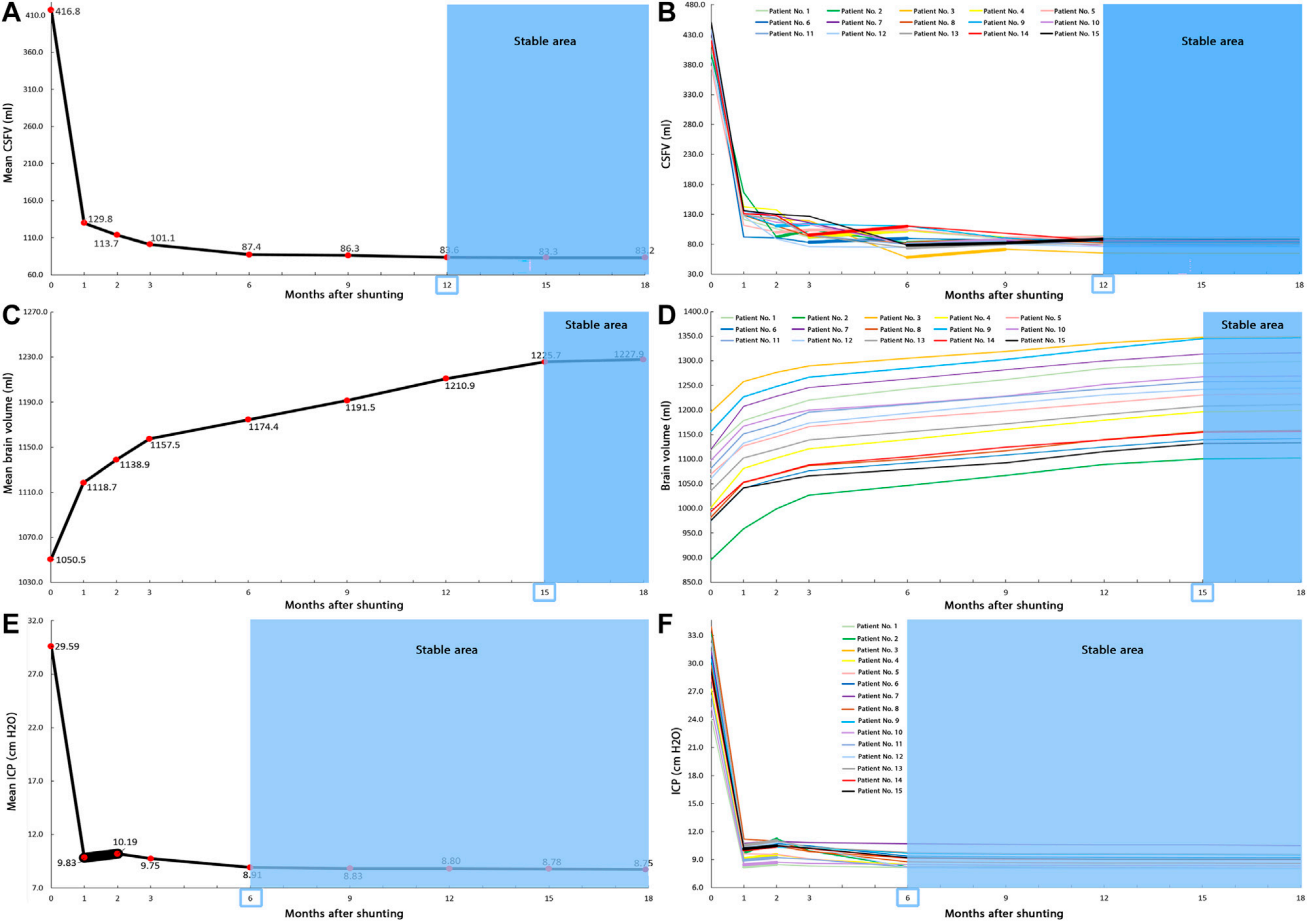
Changes of CSFV, brain volume, and ICP over 18 months after shunting. **(A)** The “mean value” of CSFV prior to shunting until 18 months after shunting. The decrease in “mean value” of CSFV from prior to shunting to 18 months after shunting was 80.0%. **(B)** CSFV changed in 15 patients prior to shunting until 18 months after shunting. Between the third and 12th months, patients had some increases in CSFV. After 12 months, CSFV reached a stable condition. **(C)** The “mean value” of the brain volume prior to shunting until 18 months after shunting. The increase in “mean value” of the brain volume from prior to shunting to 18 months after shunting was 16.9%. **(D)** The brain volume changes of 15 patients prior to shunting until 18 months after shunting. In all steps, there was an increase in the brain volume of all patients. After 15 months, the brain volume reached a stable condition. **(E)** The “mean value” of ICP prior to shunting until 18 months after shunting. The decrease in “mean value” of ICP from prior to shunting to 18 months after shunting was 70.4%. **(F)** ICP changes of 15 patients prior to shunting until 18 months after shunting. In the second month, 12 patients had an increase in ICP value. After 6 months, ICP reached a stable condition. The bold lines indicate the ascending trend, and blue areas show the stable areas of CSFV, brain volume, and ICP changes. Raw data for Figures 2A–C are included in Supplementary Tables S1, S2, and S3, respectively. CSFV: cerebrospinal fluid volume; and ICP: intracranial pressure.

CSF is an incompressible fluid, and its volume is constant. However, the volume of CSF can increase due to the accumulation of excess CSF as a result of an imbalance between production and absorption. Hence, CSF cannot be the reason for its volume change; its volume change depends on other parameters. The results of Figure 3A showed that the “mean value” of CSFV in all 15 patients during the 18 months had a *descending* trend. The decreases in “mean value” of CSFV in 1, 2, 3, 6, 9, and 12 months after shunting were 68.9, 72.7, 75.7, 79.0, 79.3, and 79.9%, respectively (Figure 3A). In the 12th month, these decreases reached a stable condition; there was no considerable change (<0.91%) in CSFV between 12 and 18 months after shunting (Figures 3A,B: blue areas, Supplementary Table S1). Tulie et al. also showed that the ventricular size of pediatric hydrocephalus patients changes until 22 months after shunting, and these changes are considerable until 12 months (Tuli et al., 1999). It should be noted that in this study, the “mean value” of CSFV prior to shunting was 416.8 ± 4.8 ml and this value in the 18th month by 80.0% decrease reached 83.2 ± 1.8 ml (Figure 3A, Supplementary Table S1).

Contrary to the *descending* trend in “mean value” of CSFV, there was an *increase* in CSFV of patient Nos. 2 (12.8%), 5 (3.7%), and 9 (2.3%) in the third month after shunting compared with that in the second month (Figure 3B, bold lines). This *increase* was also seen in patient Nos. 1 (12.3%), 4 (11.4%), 5 (0.7%), 6 (7.5%), and 14 (15.8%) in the sixth month after shunting compared with that in the third month (Figure 3B, bold lines). This *ascending* trend had been established for patient Nos. 2 (5.6%), 3 (23.2%), 7 (6.2%), 8 (6.3%), 10 (9.7%), 11 (4.6%), 12 (6.6%), 13 (9.2%), and 15 (4.7%) in the ninth month after shunting compared with that in the sixth month (Figure 3B, bold lines). This *increase* in CSFV was also in patient Nos. 1 (4.1%), 2 (3.3%), 5 (0.9%), 7 (1.3%), and 15 (7.7%) in the 12th month after shunting compared with that in the ninth month (Figure 3B, bold lines). The coefficient of variation (a statistical index to describe the dispersion of a dataset) for the CSFV data between the third and 12th months was much higher than that between the first two months (Supplementary Table S1). This parameter also confirms overdispersion and instability in CSFV data between the third and 12th months.

### Brain Volume Change

Following the increase in ICP around brain tissue, the brain volume is directly changeable. Other variables such as the biochemical process in the brain also maintain the brain’s functions and control the brain volume. From the macroscopic approach for biofluid transport, CSF (as a fluid) cannot have an entirely independent characteristic compared with the brain. This means that contrary to the CSF, brain tissue may be the reason for its volume change; therefore, its volume can change independently. The results of Figure 3C show that the “mean value” of the brain volume had an *ascending* trend. The increase in brain volume from 15 to 18 months after shunting was negligible (<0.24%) (Figures 3C,D, blue area, Supplementary Table S2); hence, follow-up of patients was stopped in the 18th month. The “mean value” of the brain volume prior to shunting was 1,050.5 ± 20.8 ml. This value increased by 16.9% to 1,227.9 ± 20.4 ml 18 months after shunting (Figure 3C, Supplementary Table S2). The brain volume ascended change in all 15 patients for 18 months (Figure 3D).

### Intracranial Pressure Change

CSF pressure in all areas of the ventricular system and SAS is calculated using FSI simulation. The pressure change in the denominator of the ICC formula is considered a CSF pressure in the upper convexity of the brain in SAS, which is defined as ICP. It should be noted that to calculate a pressure value, the software uses the first node of the element as a reference point (ADINA, 2005). The FSI simulation results showed that there were no differences between pulsatile ICP diagrams in the fourth and fifth cardiac cycles. Hence, the maximum ICP in the fourth cycle was reported for each patient in each step. The definition of “mean value” of ICP is the average of maximum ICP in the fourth cycle of all patients in that specific stage. The results of “mean value” of ICP and ICP up to 18 months after shunting are shown in Figure 3Eand Figure 3F, respectively.

One month after shunting, the “mean value” of ICP from 29.59 ± 0.8 cm H_2_O decreased significantly (66.8%) to 9.83 ± 0.2 cm H_2_O (Figure 2E). Contrary to the descending trend in “mean values” of CSFV, the “mean value” of ICP in the second month after shunting had an *increase* as compared to that in the first month and reached 10.19 ± 0.2 cm H_2_O (Figure 3E, Supplementary Table S3). This trend in the other months was *descending* and reached a stable condition in the sixth month after shunting (Figure 3E, blue areas) since there was a less than 1.98% difference in ICP values between 6 and 18 months after shunting in all 15 patients (Figure 3F, Supplementary Table S3). The “mean value” of ICP in the 18th month after shunting reached 8.75 ± 0.2 cm H_2_O, a 70.4% decrease compared with the “mean value” of ICP prior to shunting (Figure 3E, Supplementary Table S3).

## Discussion

Anile et al. proved that ICC is a time-dependent parameter (Anile et al., 2010); hence, the elapsed time between the measurement of two values (V_1_ and V_2_ or P_1_ and P_2_) is of great importance. Tuli et al. showed that the ventricular size (the numerator of the ICC equation) may change in pediatric hydrocephalus patients until 22 months after shunting (Tuli et al., 1999). Our previous studies showed that the ICP (the denominator of the ICC equation) may change in patients with noncommunicating hydrocephalus patients until 30 months after shunting (Gholampour et al., 2017a; Gholampour, 2018). Therefore, the present study suggests a new definition for the concept of ICC (long-term ICC). In this new definition, the elapsed time to calculate CSFV and ICP changes and consequently long-term ICC is 18 months. The elapsed time in the previous ICC definitions (short-term ICC) was less than a couple of seconds (by *in vitro* or lumped models (Baghbani, 2019; Gholampour and Bahmani, 2021)) or a couple of minutes/hours (by CSF infusion or bolus injection methods (Katzman and Hussey, 1970; Whittle, 2000; Fukuhara et al., 2001; Eide, 2017)). The process of ICC estimation indirectly based on the ICP pulse waveform was also less than a couple of seconds or minutes (Brasil et al., 2021). It is worth mentioning that the effect and importance of elapsed time on the CSFV–ICP graph and ICC trend were shown even in previous short-term ICC studies (Salman, 1997; Greitz, 2004; Kazimierska et al., 2021).

**FIGURE 4:**
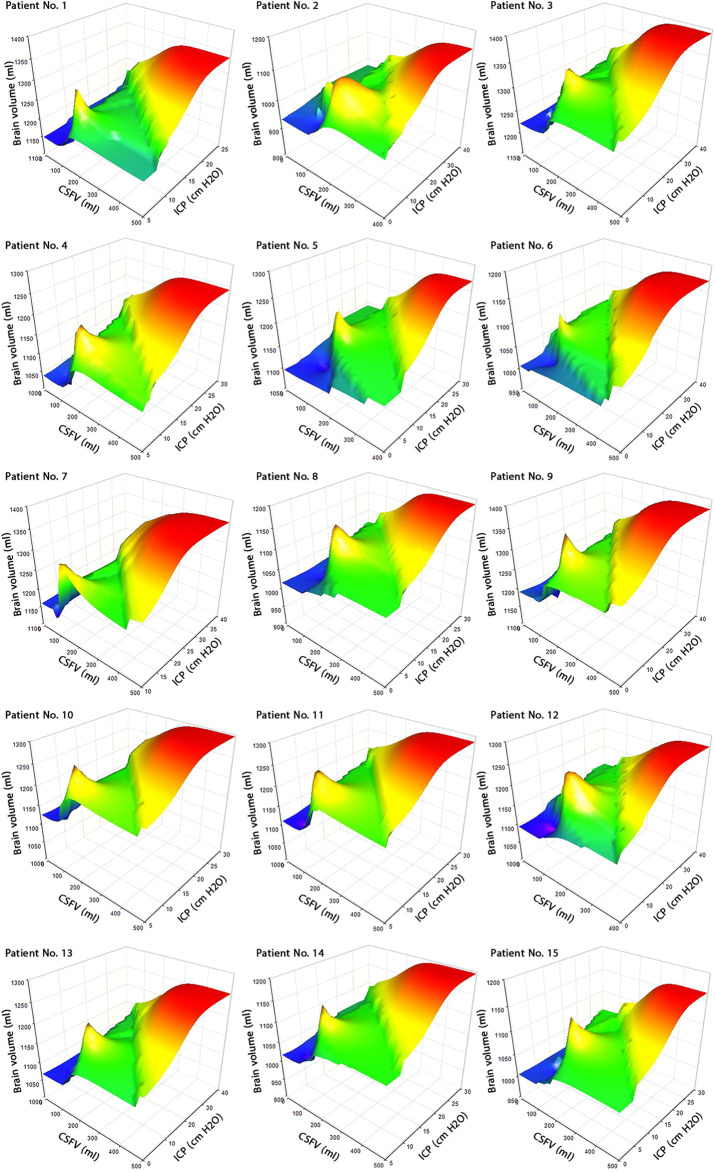
Concurrent changes of CSFV, brain volume, and ICP. 3D graphs of CSFV–brain volume–ICP during shunt treatment for all 15 patients. The changes in CSFV, brain volume, and ICP from prior to shunting to 18 months after shunting were not uniform, and the surfaces of graphs are not smooth. Raw data for Figure 3 are included in Supplementary Tables S1, S2, and S3. CSFV: cerebrospinal fluid volume; and ICP: intracranial pressure.

The results of long-term ICC calculations showed that the effect of shunt treatment on volume changes of the brain and CSF was not the same (Figure 4). Although the “mean values” of CSFV in all months had a *descending* trend (Figure 3A), this trend in some patients between 3 and 12 months after shunting was *ascending* (Figure 3B, bold lines, Figure 4). Contrary to the CSFV, the trend of brain volume changes of each patient obeyed the general trend of “mean value” changes of the brain volume, and all of them had a uniform (*ascending*) trend (Figures 3C,D). The Monro–Kellie doctrine would predict that under normal conditions, the total volumes of the CSF, brain, and cerebral blood are constant values in the long term. However, recent observations have shown that the Monro–Kellie doctrine is likely not correct (Mascarenhas et al., 2012; Klostranec et al., 2021). It can be still deduced from this doctrine that the cerebral blood volume involves the volume changes of the brain and CSF. Regarding the uniform trend in the brain volume change, a part of the nonuniformity and instability of CSFV changes (Figure 3B) might have been carried by volume changes of the cerebral blood. Based on the glymphatic theory, the volume change of the interstitial fluid may also have had a non-negligible role in carrying this nonuniformity and instability in CSFV change.

Despite few changes in the brain volume (16.9%), there was a considerable change in the ICP level (70.4%) after 18 months of shunting (Figures 3C–F). On the contrary, ICP reached a stable condition after 6 months, while brain volume was stable after 15 months (Figures 3C–F, blue areas). The longer required time for brain volume to reach a stable condition can be related to the “viscous” component in the brain tissue. The most important role of the “viscous” component is to dampen the loading or reloading effects (reloading over-ICP due to hydrocephalus by shunting) to relax the brain tissue and reach a stable condition. The “viscous” component of the brain tissue is a time-dependent parameter, which can lead to a longer required time in brain volume to reach a stable condition.

It should be noted that the “mean value” of ICP (Figure 3E, bold lines), and ICP values in 12 of 15 patients (Figure 3F, bold lines, Figure 4) had an *increase* in the second month after shunting. The excess CSF due to hydrocephalus was drained immediately after shunting. Due to the load history-dependent behavior and time-dependent characteristic of the “viscous” component (Woo et al., 1981) in the brain, the brain tissue cannot adapt itself to this significant and fast ICP reduction due to shunting immediately. Hence, following a 66.8% decrease in the “mean value” of ICP due to shunting after the first month, ICP increased by 3.7% in the second month (Figure 3E). In the third month after shunting, the “mean value” of ICP returned to the level of the first month with an error of less than 0.8% (Figure 3E). It can be deduced that due to its “viscous” component, the brain tissue needed approximately two months (between the first and third months) to adapt itself to the fast and significant ICP reduction due to shunting.

**FIGURE 5:**
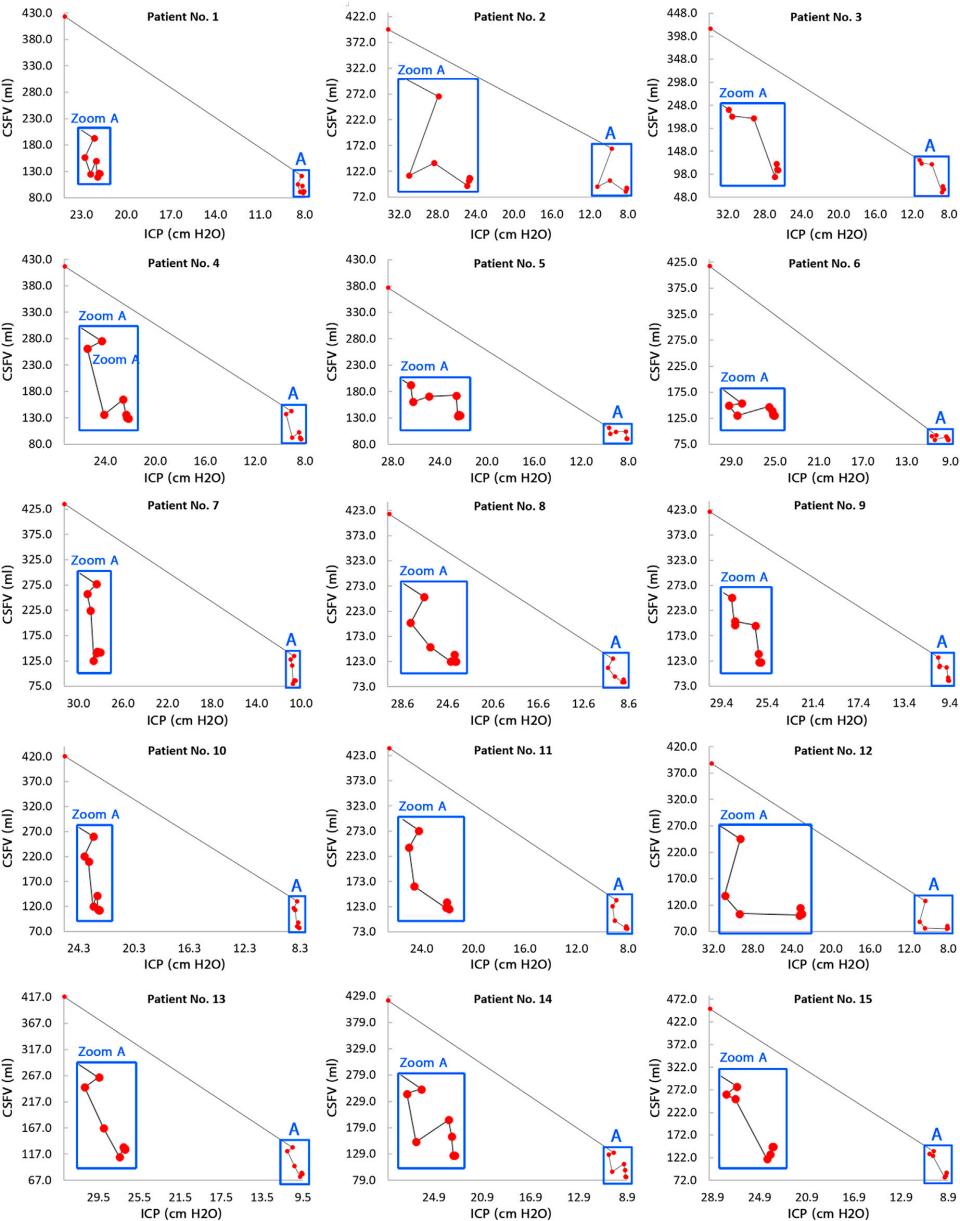
CSFV–ICP graphs for all 15 patients. The general trend of the CSFV–ICP graphs in all 15 patients was not monotonic. The slopes of these graphs are representative of the long-term ICC changes during shunt treatment. There are 9 red circles in each panel with each circle representing a step. The steps include prior to shunting, and 1, 2, 3, 6, 9, 12, 15, and 18 months after shunting. In each panel, the first red circle on the left side represents prior to shunting, and the last red circle on the right side represents 18 months after shunting; hence, moving from the left to the right shows the progression of the treatment process. Raw data for Figure 4 are included in Supplementary Tables S1 and S3. CSFV: cerebrospinal fluid volume; ICP: intracranial pressure; and ICC: intracranial compliance.

**FIGURE 6:**
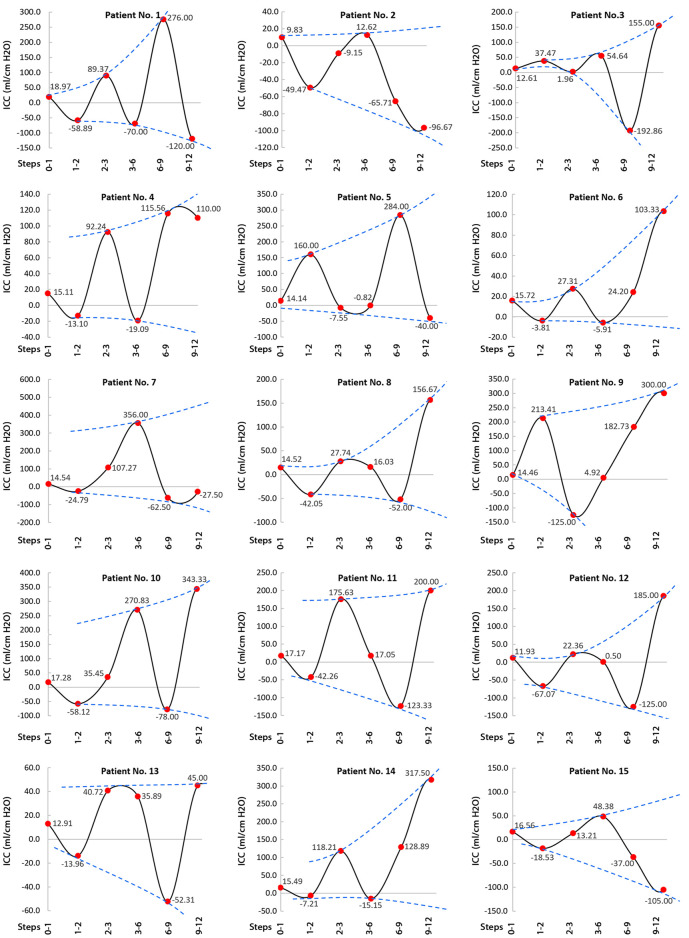
Long-term ICC changes from prior to shunting to 12 months after shunting. The long-term ICC changes between prior to shunting and the first month after shunting are shown by the first red circle on the left side of each panel. The definitions of steps 0-1, steps 1-2, steps 2-3, steps 3-6, steps 6-9, and steps 9-12 in horizontal axes are the long-term ICC values between prior to shunting and 1 month after shunting, between 1 and 2 months after shunting, between 2 and 3 months after shunting, between 3 and 6 months after shunting, between 6 and 9 months after shunting, and between 9 and 12 months after shunting, respectively. It should be noted that after 6 months, the ICP changes (numerator of ICC equation) and CSFV changes (denominator of ICC equation) were 1.98 and 4.70%, respectively. Hence, despite an oscillatory increase in long-term ICC values over the course of 12 months, this increase until 6 months after shunting (0-1, 1-2, 2-3, and 3-6 steps) is more referable for our analysis. The dashed blue curves in each panel are the envelope curves of the long-term ICC graph. These envelope curves in all panels show a nonuniform and oscillatory increase in long-term ICC values during the treatment process (from the left to right). Raw data for Figure 5 are included in Supplementary Table S4. CSFV: cerebrospinal fluid volume; ICP: intracranial pressure; and ICC: intracranial compliance.

CSFV and ICP values were calculated over the course of 18 months (in 9 stages) to draw the CSFV–ICP graph for each patient (Figure 5) that the “slope” of this graph represents ICC (Figure 6). The ICC calculation process is described in Supplementary Table S4. ICC was calculated based on the definition of “slope” in the CSFV–ICP graph, and ICP and CSFV between every two stages in a row were used as reference points to calculate ICC. The ICC trend prior to shunting to the first month after shunting (steps 0–1) under the new definition (long-term ICC) descended for all patients with a “mean value” of 14.75 ± 0.6 ml/cm H_2_O (Supplementary Table S4). However, the ICC value in previous studies (short-term ICC) was less than 0.57 ml/cm H_2_O (Sahuquillo et al., 1991; Eide, 2017).

The previous CSFV–ICP graphs were separated into two compensatory reserve zones: the first zone with a lower ICC value and the second zone with a higher ICC value (Czosnyka et al., 2004; Okon et al., 2018; Heldt et al., 2019). However, compensatory reserve behavior in the corresponding graphs in the present study was variable and not necessarily separated in some ICC zones (Figure 6). ICC values during shunt treatment had a nonharmonic oscillatory behavior (Figure 6). Following the ICP reduction during shunt treatment, ICC increased with an oscillatory and nonuniform trend. The envelope curves of the ICC graphs (Figure 6, blue dash lines) showed an ascending trend in ICC amplitude during the shunt treatment process (from the left side to the right side in the planes of Figure 6). In previous studies, short-term ICC increased, but the increase in ICC in the present study under the new definition (long-term ICC) was oscillatory and nonuniform (Figure 5, zoom A, and Figure 6). It should be emphasized that Figure 6 shows that the ICC fluctuates until 12 months after shunting. ICP reached a stable condition after 6 months, and both CSFV and ICP had a stable condition after 12 months. Hence, the differences between CSFV and ICP changes after 12 months were negligible, and the trend of ICC was not ascending and/or meaningful.

Previous studies showed the potential impact of brain biomechanics/intracranial compliance as an emerging concept in the pathogenesis of hydrocephalus and other disorders due to altered ICP (Duy et al., 2022a; Duy et al., 2022b). There was also a widely held belief in previous studies that the short-term CSFV–ICP graph is a *monoexponential* function (Marmarou et al., 1975; Sklar and Elashvili, 1977; Szewczykowski et al., 1977; Avezaat et al., 1979; Shapiro et al., 1980; Raksin et al., 2003; Gholampour and Bahmani, 2021). The present study under the new definition (long-term) showed that the CSFV–ICP graphs in all patients were *nonmonotonic* (Figure 5) and the trends of ICC changes were oscillatory (nonuniform) (Figure 6). We did not calculate the CSFV–ICP graph and short-term ICC using our method (calculation of ICP and consequently ICC using FSI simulation) because the goal of the present study was to assess ICC in the long term. However, the results of the study by Okon et al. in the short term also showed the trend of the CSFV–ICP graph was *nonmonotonic* (Okon et al., 2018). They used the lumbar puncture to drain CSF, and their measured ICC reached 2.5 ± 1.35 ml/cm H_2_O*.* In previous studies, this value was less than 0.57 ml/cm H_2_O (Sahuquillo et al., 1991; Eide, 2017).

On the contrary, one of the most important findings of that widely held belief about *monoexponential* shape for short-term ICC graph was the claim that when following a *decrease* in the ICP level during the treatment process of hydrocephalus patients, ICC will certainly *increase* (Marmarou et al., 1975; Shapiro et al., 1980). Our recent *in vitro* study for very short-term ICC measurement (Gholampour and Bahmani, 2021) and other studies that estimated ICC indirectly in a very short time (Bateman, 2000; Balédent et al., 2004; Wagshul et al., 2006) also confirmed this claim. However, the results of the present study over a long period of time showed that when following a *decrease* in ICP values during the treatment process, ICC did not necessarily *increase* (Figure 5, zoom A) and ICC values had an oscillatory increase (Figure 6). Previously, indirect ICC estimations using *in vivo* methods that confirmed increasing ICC with a decrease in ICP during a very short time (in a couple of seconds (Brasil et al., 2021)) might have been associated with the risk of measurement error of ICC due to the preconditioning property of the brain tissue. The study by Fukuhara et al. measured short-term ICC in obstructive dogs with hydrocephalus using the saline infusion at two ICP levels (Fukuhara et al., 2001). They increased the ICP level to 15–25 mm Hg after shunting. Then, ICP *decreased* by 37.35% after two weeks, and ICC also *decreased* significantly. In total, their results support our idea that a long-term evaluation of ICC would be essential in the assessment of hydrocephalus as the results confirmed the changes in the slope of the CSFV–ICP graphs and ICC values after shunting.

**FIGURE 7:**
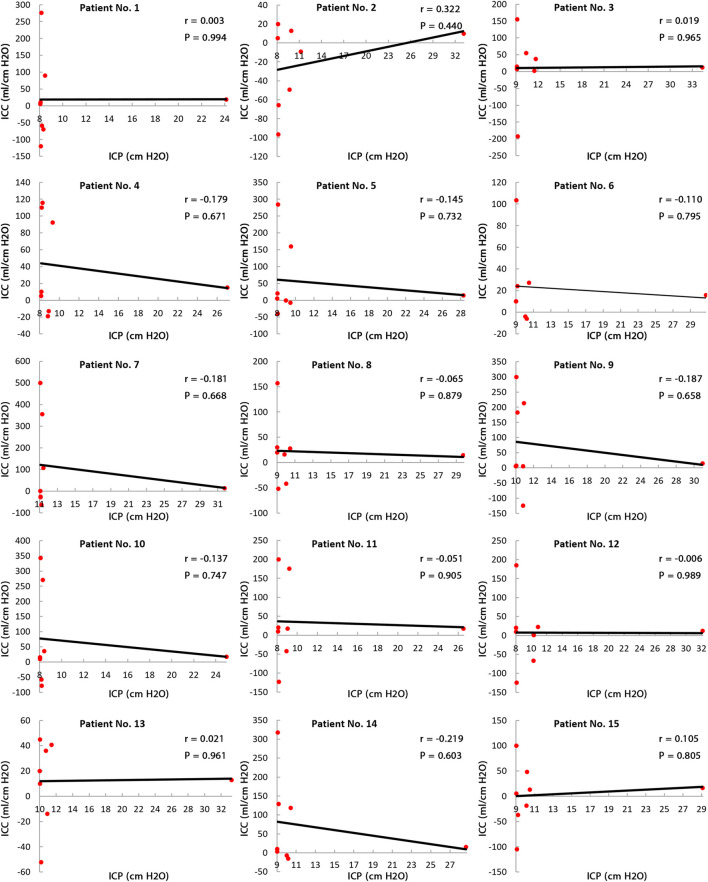
Relationship between long-term ICC and ICP. Pearson’s correlation results between long-term ICC and ICP in all 15 patients show that all *ps* are greater than 0.44, so the correlations are not statistically “significant.” This means there is no *linear* relationship between long-term ICC and ICP. Raw data for Figure 6 are included in Supplementary Tables S3 and S4. ICP: intracranial pressure; and ICC: intracranial compliance.

Eide’s use of the CSF infusion method over a short time period showed that there was not a *linear* relationship between short-term ICC and ICP in adult hydrocephalus patients (Eide, 2017). Our Pearson’s correlation analysis in Figure 7 confirms the results of the study by Eide and showed that the relationship between long-term ICC and ICP was not statistically significant (all *p* >0.44) in any of the 15 patients. Therefore, there is not a *linear* relationship between long-term ICC and ICP. It should be noted that the lack of this *linear* relationship between ICC and ICP can be a basis to interpret the negligible ICP change in normal-pressure hydrocephalus patients. With hydrocephalus occurring with likely ICC changes in these patients, ICP has no significant change.

The present study (under the new definition) showed that there are variations and nonuniformities in the CSFV (between the third and the 12th month) and the ICP level (the second month) (Figures 3B,F, bold lines). These results also showed that 6 months is needed to reach a plateau and stable condition when the over-ICP due to hydrocephalus is relieved by shunting (Figure 3F, blue area). The CSFV–ICP graph under the new definition was nonmonotonic (Figure 5, zoom A), while this graph in previous ICC studies (short-term ICC) was monotonic (a *monoexponential* function). The trend of long-term ICC changes was nonuniform and oscillatory (Figure 6), while these changes under the previous short-term ICC were uniform and include two zones. Similar to the previous study that was used for short-term ICC, the relationship between long-term ICC and ICP in the present study under the new definition was not *linear* (Figure 7). Furthermore, the *monoexponential* shape of the CSFV–ICP graph under the previous definition was not relevant to the type of disease (Wiegand and Richards, 2007; Heldt et al., 2019), though this may not be a unique and independent characteristic for hydrocephalus patients. For example, the inverse relationship between short-term ICC and ICP, and the *monoexponential* trend in the CSFV–ICP graph were established in other patients, such as those with head injuries and brain edema (Maset et al., 1987; Jeong, 2016). Our method based on the new definition cannot be used for patients with other diseases because our method can only work when there is a natural volume and/or pressure change. Hence, we cannot compare the sensitivity of our method with other diseases.

### Open Questions and Future Works

A. Patients’ medical histories showed they had a permanent health condition in the 18th month with significant clinical fluctuations, variations, and nonuniformities in their clinical conditions during these 18 months. Perhaps the nonuniform and oscillatory behavior of long-term ICC graphs in Figure 6 is representative of these clinical variations. One needs to evaluate this in future studies to assess the relationship between oscillatory (nonuniform) changes in long-term ICC trends (negative and positive values in ICC diagram (Figure 6)) as a numerical index to reflect the clinical nonuniformities and variations of hydrocephalus patients after shunting. It should be noted that negative and positive slopes in CSFV–ICP graph (long-term ICC) were also observed in the short-term ICC of the study by Okon et al. (2018).

B. The majority of previous short-term ICC measurements were carried out using the bolus injection or CSF infusion (Marmarou method). The Marmarou method may lead to disruption in the function of the CSF production and absorption (glymphatic system) mechanism because of additional injected artificial CSF. Hydrocephalus may be due to an absorption impairment; however, this rarely accompanies the overproduction of CSF as seen in injected CSF in the Marmarou method. The present method has not manipulated the natural mechanism of shunted hydrocephalus patients by adding external artificial CSF in the natural system of CSF circulation. Also, there were not any interventions in the physiological system of the CSF circulation of shunted hydrocephalus in the present study. Neurosurgeons changed the shunt opening pressure of 24 patients for 18 months, while the 15 patients assessed in the present study had successful shunting without changes in the valve opening pressure. Okon et al. also did not manipulate the natural mechanism of the CSF circulation system in their short-term ICC measurement method and only used lumbar puncture instead of the shunt to drain excess CSF in patients (Okon et al., 2018). Their results showed a *nonmonotonic* behavior in short-term CSFV–ICP trend, which is similar to our study, and in contrast to previous studies. Our results also support the long-time effects of “viscous” component of brain tissue in the assessment of hydrocephalus patients after shunting. Future investigators can use the Marmarou method to measure short-term ICC after shunting for comparison with our results. However, it can be deduced that without considering the follow-up conditions of the Marmarou method, the effect of *time-*dependent characteristics (the “viscous” component) of brain tissue may not be shown in short-term ICC measurements (Marmarou method). The Marmarou method was able to increase our understanding of complexities in hydrocephalus for half a century; however, whether ICC measurements over a short time are an advantage for the Marmarou method or a disadvantage has yet to be determined.

C. The words “brain compliance” and “intracranial compliance (ICC)” were used interchangeably in many studies. It would be better to define the numerator and denominator of the “brain compliance” formula as the brain volume and cerebral perfusion pressure (CPP) change. CPP is defined as the difference between mean arterial pressure and ICP. Calculating this parameter is important in analyzing the brain behavior after shunting. For a comprehensive evaluation of hydrocephalus patients during the shunt treatment process, it is suggested that “brain compliance” be calculated under the new definition (long-term measurement).

D. We suggest comparing the long-term ICC trend in future studies with a larger population by considering patients with all types of hydrocephalus such as noncommunicating and normal pressure hydrocephalus and for pediatric and adult patients. In addition, expanding the concept of long-term compliance for the spinal canal as long-term spinal compliance may also be interesting to study in future works.

## Conclusions

Our results for long-term ICC calculation showed that the CSFV–ICP graph is nonmonotonic, ICC change is nonuniform, the relationship between ICC and ICP is not *linear*, and ICC increased oscillatory behavior during the shunt treatment of communicating hydrocephalus patients. The results of the present noninvasive study may be useful for future studies to link the clinical nonuniformities and variations of these patients after shunting with nonuniform and oscillatory changes of the long-term ICC.

## Data Availability

The raw data supporting the conclusions of this article will be made available by the authors, without undue reservation.

## Abbreviations

ICC, intracranial compliance; CSF, cerebrospinal fluid; CSFV, cerebrospinal fluid volume; ICP, intracranial pressure; FSI, fluid–structure interaction; CINE PC-MRI, cine phase-contrast magnetic resonance imaging; CFD, computational fluid dynamics; SAS, subarachnoid spaces; and CPP, cerebral perfusion pressure.
