# Supplementary material for: A New Definition for Intracranial Compliance to Evaluate Adult Hydrocephalus After Shunting
Source: Front Bioeng Biotechnol. 2022 Aug 1;10:900644. doi: 10.3389/fbioe.2022.900644 (PMC9377221; doi:10.3389/fbioe.2022.900644)
Supplement: Supplementary file 1 [file DataSheet1.PDF]

A new definition for intracranial compliance to evaluate adult hydrocephalus after shunting

Seifollah Gholampour, Bakhtiar Yamini[1], Julie Droessler[1], David Frim[1*]

[1] Department of Neurological Surgery, University of Chicago, Chicago, Illinois, USA.

***Correspondence:** David Frim
Department of Neurological Surgery
The University of Chicago
5841 S. Maryland Ave, 60637, Chicago, IL, USA.
Tel: (+1) 773-702-2123
Fax: (+1) 773-702-3518
E-mail: dfrim@bsd.uchicago.edu
ORCID: 0000-0001-7219-2249

Short Title: A new definition for intracranial compliance

Number of Supplementary table: 4
Number of Supplementary figure: 0

Supplementary Table 1. Statistical analysis of CSFV changes for all 15 patients in 9 steps: before shunting, and 1, 2, 3, 6, 9, 12, 15, and 18 months after shunting. It should be noted that the values in the table are CSFV (ml).

CSFV: cerebrospinal fluid volume, SD: standard deviation, SE: standard error, CV: coefficient of variation, CI: confidence of interval.

Patient No. / Month after shunting	1	2	3	4	5	6	7	8	9	10	11	12	13	14	15	Mean	SD	SE	CV	CI level	95% CI	
																					Lower	Upper
0	423.5	396.7	414.9	416.7	376.8	417.5	434.8	415.7	419.7	420.8	437.6	388.2	417.8	420.6	450.6	416.8	18.5	4.8	4.4	10.3	406.5	427.1
1	121.5	166.2	128.6	142.8	111.3	92.2	134.8	128.7	130.1	130.4	135.7	127.5	129.5	131.4	136.5	129.8	15.7	4.0	4.9	8.7	121.1	138.5
2	105.6	92.0	121.5	137.3	100.1	90.6	127.6	110.2	110.9	116.5	123.4	88.6	122.4	128.3	130.2	113.7	15.5	4.0	13.7	8.6	105.1	122.3
3	91.3	103.8	119.3	92.1	103.8	83.5	115.8	93.0	113.4	112.6	95.3	76.3	94.3	95.2	126.5	101.1	14.1	3.6	13.9	7.8	93.3	108.9
6	102.5	82.6	58.1	102.6	104.5	89.8	80.2	82.1	110.3	80.1	80.3	75.2	74.2	110.2	78.6	87.4	15.3	3.9	17.5	8.5	79.0	95.9
9	88.7	87.2	71.6	92.2	90.3	87.1	85.2	87.3	90.2	87.9	84.0	80.2	81.0	98.6	82.3	86.3	6.2	1.6	7.1	3.4	82.8	89.7
12	92.3	90.1	65.4	90.0	91.1	84.0	86.3	82.6	84.2	77.6	80.0	76.5	80.1	85.9	88.6	83.6	7.1	1.8	8.4	3.9	79.7	87.6
15	92.1	89.7	65.1	89.6	90.7	83.7	86.1	82.3	83.9	77.3	79.6	76.1	79.8	85.7	88.5	83.3	7.1	1.8	8.5	3.9	79.4	87.3
18	92.0	89.6	64.8	89.5	90.6	83.5	86.0	82.1	83.8	77.1	79.3	76.0	79.4	85.6	88.4	83.2	7.1	1.8	8.6	4.0	79.2	87.1

Supplementary Table 2. Statistical analysis of the brain volume changes for all 15 patients in 9 steps: before shunting, and 1, 2, 3, 6, 9, 12, 15, and 18 months after hunting. It should be noted that the values in the table are the brain volume (ml).

SD: standard deviation, SE: standard error, CV: coefficient of variation, CI: confidence of interval.

Patient No. / Month after shunting	1	2	3	4	5	6	7	8	9	10	11	12	13	14	15	Mean	SD	SE	CV	CI Level	Lower	Upper
0	1118.5	895.5	1195.4	1001.5	1070.0	974.4	1119.0	982.2	1156.3	1097.1	1081.0	1061.1	1036.2	994.0	975.8	1050.5	80.6	20.8	7.7	1005.9	1095.2	44.6
1	1178.6	958.3	1257.9	1081.4	1126.7	1041.2	1207.8	1052.7	1226.7	1166.7	1151.4	1132.5	1102.5	1053.7	1041.7	1118.7	82.0	21.2	7.3	1073.3	1164.1	45.4
2	1199.4	999.2	1276.5	1102.4	1146.1	1060.4	1228.1	1069.8	1247.8	1185.5	1169.9	1153.7	1120.1	1070.3	1054.2	1138.9	80.1	20.7	7.0	1094.5	1183.3	44.4
3	1220.1	1027.3	1289.8	1121.0	1165.8	1076.5	1245.7	1086.6	1266.4	1199.6	1195.6	1173.4	1139.2	1088.4	1066.4	1157.5	79.2	20.5	6.8	1113.6	1201.3	43.9
6	1242.6	1046.6	1305.5	1140.3	1183.5	1092.6	1263.1	1099.8	1284.4	1213.1	1211.0	1193.2	1155.5	1105.1	1079.3	1174.4	79.7	20.6	6.8	1130.2	1218.5	44.2
9	1261.4	1067.1	1319.4	1160.3	1198.4	1108.2	1281.7	1117.4	1302.3	1227.9	1227.4	1212.5	1171.5	1124.5	1092.3	1191.5	79.4	20.5	6.7	1147.5	1235.5	44.0
12	1284.5	1089.6	1336.5	1179.6	1214.5	1124.5	1299.5	1139.7	1324.6	1251.8	1242.8	1230.7	1190.7	1139.4	1115.1	1210.9	79.3	20.5	6.5	1167.0	1254.8	43.9
15	1295.5	1100.5	1347.8	1196.3	1230.4	1139.3	1313.3	1156.3	1345.2	1267.4	1257.5	1242.2	1208.2	1154.7	1131.5	1225.7	79.2	20.4	6.5	1181.9	1269.6	43.9
18	1298.3	1102.1	1349.3	1199.2	1232.9	1141.9	1316.4	1158.7	1346.4	1269.2	1258.7	1244.5	1211.1	1156.0	1133.8	1227.9	79.1	20.4	6.4	1184.1	1271.7	43.8

Supplementary Table 3. Statistical analysis of ICP changes for all 15 patients in 9 steps: before shunting, and 1, 2, 3, 6, 9, 12, 15, and 18 months after hunting. It should be noted that the values in the table are ICP (cm H_2O).

ICP: intracranial pressure, SD: standard deviation, SE: standard error, CV: coefficient of variation, CI: confidence of interval.

Patient No. / Month after shunting	1	2	3	4	5	6	7	8	9	10	11	12	13	14	15	Mean	SD	SE	CV	CI Level	95% CI	
																					Lower	Upper
0	24.13	33.16	33.89	27.24	28.39	30.97	31.26	29.77	30.45	25.27	26.51	32.14	32.85	28.68	29.11	29.59	2.9	0.8	9.9	1.6	28.0	31.2
1	8.21	9.71	11.18	9.11	9.62	10.28	10.63	10.01	10.42	8.47	8.92	10.29	10.52	10.01	10.14	9.83	0.8	0.2	8.5	0.5	9.4	10.3
2	8.48	11.21	10.99	9.53	9.55	10.70	10.92	10.45	10.33	8.71	9.21	10.87	11.03	10.44	10.48	10.19	0.9	0.2	8.6	0.5	9.7	10.7
3	8.32	9.92	9.87	9.04	9.06	10.44	10.81	9.83	10.31	8.60	9.05	10.32	10.34	10.16	10.20	9.75	0.7	0.2	7.7	0.4	9.3	10.2
6	8.16	8.24	8.75	8.49	8.21	9.38	10.71	9.15	9.68	8.48	8.17	8.10	9.78	9.17	9.21	8.91	0.8	0.2	8.5	0.4	8.5	9.3
9	8.11	8.17	8.68	8.40	8.16	9.27	10.63	9.05	9.57	8.38	8.14	8.06	9.65	9.08	9.11	8.83	0.7	0.2	8.4	0.4	8.4	9.2
12	8.08	8.14	8.64	8.38	8.14	9.24	10.59	9.02	9.55	8.35	8.12	8.04	9.63	9.04	9.05	8.80	0.7	0.2	8.4	0.4	8.4	9.2
15	8.06	8.12	8.62	8.34	8.12	9.21	10.59	9.01	9.51	8.33	8.10	8.02	9.60	9.02	9.05	8.78	0.7	0.2	8.4	0.4	8.4	9.2
18	8.04	8.10	8.58	8.32	8.10	9.19	10.50	9.00	9.49	8.31	8.07	8.01	9.58	8.99	9.03	8.75	0.7	0.2	8.3	0.4	8.3	9.2

Supplementary Table 4. ICC calculation process and statistical analysis of ICC changes for all 15 patients in the steps: Between before shunting and 1 month after shunting (0-1), between 1 and 2 months after shunting (1-2), between 2 and 3 months after shunting (2-3), between 3 and 6 months after shunting (3-6), between 6 and 9 months after shunting (6-9), and between 9 and 12 months after shunting (9-12). It should be noted that after 12 months after shunting, CSFV also reached a stable condition similar to ICP which had reached to stable condition after six months. Hence, the calculation process of ICC is stopped. The unites of CSFV, ICP, and ICC in the table are ml, cm H_2O, and ml/cm H_2O, respectively.

CSFV: cerebrospinal fluid volume, ICP: intracranial pressure, ICC: intracranial compliance, SD: standard deviation, SE: standard error, CV: coefficient of variation, CI: confidence of interval.

Patient No.		1		2		3		4		5		6		7		8		9		10		11		12		13		14		15		Mean ICC	SD	SE	CV	CI level	95% CI	
**Step**	**Parameters**	**Δ ICP**	**Δ CSFV**	**Δ ICP**	**Δ CSFV**	**Δ ICP**	**Δ CSFV**	**Δ ICP**	**Δ CSFV**	**Δ ICP**	**Δ CSFV**	**Δ ICP**	**Δ CSFV**	**Δ ICP**	**Δ CSFV**	**Δ ICP**	**Δ CSFV**	**Δ ICP**	**Δ CSFV**	**Δ ICP**	**Δ CSFV**	**Δ ICP**	**Δ CSFV**	**Δ ICP**	**Δ CSFV**	**Δ ICP**	**Δ CSFV**	**Δ ICP**	**Δ CSFV**	**Δ ICP**	**Δ CSFV**						**Upper**	**Lower**
**0-1**	**Δ between the values in steps 0-1**	-15.92	-302.00	-23.45	-230.50	-22.71	-286.28	-18.13	-273.90	-18.77	-265.50	-20.69	-325.30	-20.63	-300.01	-19.76	-287.00	-20.03	-289.59	-16.80	-290.35	-17.59	-301.94	-21.85	-260.70	-22.33	-288.28	-18.67	-289.20	-18.97	-314.10							
	**ICC = ΔCSFV/ΔICP**	**18.97**		**9.83**		**12.61**		**15.11**		**14.14**		**15.72**		**14.54**		**14.52**		**14.46**		**17.28**		**17.17**		**11.93**		**12.91**		**15.49**		**16.56**		**14.75**	**2.32**	**0.60**	**15.76**	**1.29**	**16.04**	**13.46**
**1-2**	**Δ between the values in steps 1-2**	0.27	-15.90	1.50	-74.20	-0.19	-7.12	0.42	-5.50	-0.07	-11.20	0.42	-1.60	0.29	-7.19	0.44	-18.50	-0.09	-19.21	0.24		0.29	-12.26		-38.90	0.51	-7.12	0.43	-3.10	0.34	-6.30							
	**ICC = ΔCSFV/ΔICP**	**-58.89**		**-49.47**		**37.47**		**-13.10**		**160.00**		**-3.81**		**-24.79**		**-42.05**		**213.41**		**-58.12**		**-42.26**		**-67.07**		**-13.96**		**-7.21**		**-18.53**		**0.78**	**80.74**	**20.85**	**10401.3**	**44.71**	**45.49**	**-43.94**
**2-3**	**Δ between the values in steps 2-3**	-0.16	-14.30	-1.29	11.80	-1.12	-2.20	-0.49	-45.20	-0.49	3.70	-0.26	-7.10	-0.11	-11.80	-0.62	-17.20	-0.02	2.50	-0.11		-0.16	-28.10		-12.30	-0.69	-28.10	-0.28	-33.10	-0.28	-3.70							
	**ICC = ΔCSFV/ΔICP**	**89.37**		**-9.15**		**1.96**		**92.24**		**-7.55**		**27.31**		**107.27**		**27.74**		**-125.00**		**35.45**		**175.63**		**22.36**		**40.72**		**118.21**		**13.21**		**40.65**	**70.16**	**18.11**	**172.57**	**38.85**	**79.51**	**1.80**
**3-6**	**Δ between the values in steps 3-6**	-0.16	11.20	-1.68	-21.20	-1.12	-61.20	-0.55	10.50	-0.85	0.70	-1.06	6.26	-0.10	-35.60	-0.68	-10.90	-0.63	-3.10	-0.12		-0.88	-15.00		-1.10	-0.56	-20.10	-0.99	15.00	-0.99	-47.90							
	**ICC = ΔCSFV/ΔICP**	**-70.00**		**12.62**		**54.64**		**-19.09**		**-0.82**		**-5.91**		**356.00**		**16.03**		**4.92**		**270.83**		**17.05**		**0.50**		**35.89**		**-15.15**		**48.38**		**47.06**	**113.31**	**29.26**	**240.78**	**62.75**	**109.81**	**-15.69**
**6-9**	**Δ between the values in steps 6-9**	-0.05	-13.80	-0.07	4.60	-0.07	13.50	-0.09	-10.40	-0.05	-14.20	-0.11	-2.66	-0.08	5.00	-0.10	5.20	-0.11	-20.10	-0.10		-0.03	3.70		5.00	-0.13	6.80	-0.09	-11.60	-0.10	3.70							
	**ICC = ΔCSFV/ΔICP**	**276.00**		**-65.71**		**-192.86**		**115.56**		**284.00**		**24.20**		**-62.50**		**-52.00**		**182.73**		**-78.00**		**-123.33**		**-125.00**		**-52.31**		**128.89**		**-37.00**		**14.84**	**147.99**	**38.21**	**996.95**	**81.95**	**96.80**	**-67.11**
**9-12**	**Δ between the values in steps 9-12**	-0.03	3.60	-0.03	2.90	-0.04	-6.20	-0.02	-2.20	-0.02	0.80	-0.03	-3.10	-0.04	1.10	-0.03	-4.70	-0.02	-6.00	-0.03		-0.02	-4.00		-3.70	-0.02	-0.90	-0.04	-12.70	-0.06	6.30							
	**ICC = ΔCSFV/ΔICP**	**-120.00**		**-96.67**		**155.00**		**110.00**		**-40.00**		**103.33**		**-27.50**		**156.67**		**300.00**		**343.33**		**200.00**		**185.00**		**45.00**		**317.50**		**-105.00**		**101.78**	**155.39**	**40.12**	**152.67**	**86.05**	**187.83**	**15.73**
